# Child marriage in Ghana: evidence from a multi-method study

**DOI:** 10.1186/s12905-019-0823-1

**Published:** 2019-11-12

**Authors:** Babatunde Ahonsi, Kamil Fuseini, Dela Nai, Erika Goldson, Selina Owusu, Ismail Ndifuna, Icilda Humes, Placide L. Tapsoba

**Affiliations:** 1 UNFPA Ghana, P. O. Box GP, 1423 Accra, Ghana; 2 Population Council, P. O. Box CT 4906, Cantonment, Accra, Ghana; 3 The Global Cottage, Inc., Florida, USA

**Keywords:** Child marriage, Culture, Ghana

## Abstract

**Background:**

Child marriage remains a challenge in Ghana. Over the years, government and development partners have made various commitments and efforts to curb the phenomenon of child marriage. However, there is little empirical evidence on the predictors, norms and practices surrounding the practice to support their efforts, a gap this study sought to fill.

**Methods:**

The study employed a multiple-method approach to achieve the set objectives. Data from the women’s file of the 2014 Ghana Demographic and Health Survey (GDHS) was used to examine the predictors of child marriage using frequencies and logistic regression methods. Data from Key Informant Interviews (KIIs) and Focus Group Discussions (FGDs) collected in Central and Northern regions of Ghana were used to examine norms and practices surrounding child marriage using thematic analysis.

**Results:**

Two in ten (20.68%) girls in the quantitative sample married as children. The results revealed that girls who had never attended school compared to those who had ever attended school were more likely to marry as children (OR, 3.01). Compared with girls in the lowest wealth quintile, girls in the middle (OR, 0.59), fourth (OR, 0.37) and highest (OR, 0.32) wealth quintiles were less likely to marry as children. From the qualitative data, the study identified poverty, teenage pregnancy, and cultural norms such as betrothal marriage, exchange of girls for marriage and pressure from significant others as the drivers of child marriage.

**Conclusions:**

The findings show that various socio-economic and cultural factors such as education, teenage pregnancy and poverty influence child marriage. Hence, efforts to curb child marriage should be geared towards retention of girls in school, curbing teenage pregnancy, empowering girls economically, enforcing laws on child marriage in Ghana, as well as designing tailored advocacy programs to educate key stakeholders and adolescent girls on the consequences of child marriage. Additionally, there is the need to address socio-cultural norms/practices to help end child marriage.

## Background

Child marriage (or early marriage) can be defined as “both formal marriages and informal unions in which a girl lives with a partner as if married before the age of 18” [1]. Child marriage, despite recent declines is still widely practiced in many parts of the developing world [2, 3]. In developing countries (excluding China), every third young woman continues to marry as a child [4]. While age at first marriage is generally increasing around the world, in many parts of sub-Saharan Africa, a significant proportion of girls still marry before their 18th birthday [5–7].

In developing countries, it is estimated that one in seven girls marry before age 15 and 38% marry before age 18 [8]. In Ghana, 4.4 and 5.8% of women aged 15–49 married by exact age 15 in 2006 and 2011 respectively. In addition, among women aged 20–24, the proportion who married before exact age 18 was 22% in 2006 and 21% in 2011 [9, 10]. The rest of the introductory section of the paper discusses reasons and incentives for child marriage, negative effects of child marriage and legal norms in relation to child marriage.

### Reasons and incentives for child marriage

Child marriage is used as a mechanism to protect chastity as premarital sex and child bearing bring shame to the family [11]. In traditional Ghanaian societies premarital sex and child bearing is frowned upon, hence early marriage is encouraged. For instance, betrothal (in some cases, exchange of girls) is often early, sometimes before birth to ensure sex and child bearing occur within marriage [12]. The need to reinforce social ties or build alliances is another traditional factor that influences child marriage [13, 14].

The major religious traditions (Christianity and Islam) in Ghana encourage early marriage because premarital sex and child bearing are considered “immoral”. These behaviours were, and often still are, strongly prohibited and sometimes punished. Both Christianity and Islam seek to ensure that sex and child bearing occur within marriage. Hence, they tend to encourage early marriage, mostly indirectly [15]. Some Muslim groups try to ensure that births occur within marriage by compressing the gap between age at menarche and marriage [16]. While traditional and religious practices try to protect girls from pre-marital sex and child bearing, girls who fall pregnant are sometimes married off to men who impregnated them to ensure they take care of them [12].

In Ghanaian societies, marriage is very important for women’s status. Recognition and respect go hand in hand with marriage. Evidence suggest that early marriage brings some child brides respect and honor as both peers and adults in the community show them respect because they have “settled down” (married) and are seen to be responsible. Parents who have married daughters also enjoy some prestige and respect from community members [12].

Another factor that contributes to child marriage is poverty [7, 11, 17–19]. Its influence on child marriage is multi-dimensional that stems from parents’ socio-economic status and children’s demand for material goods that their parents cannot afford (in some cases attributable to parental neglect and supervision). Some parents and girls are motivated by financial gains and security to the family and they tend to agree to child marriage. In some cases, it provides financial stability to girls coming from economically disadvantaged homes as some child brides married to escape poverty. Child brides do not only get financial support from their husbands, but also from their in-laws to ensure they lack little or nothing. Some child brides are also able to amass some wealth from their husbands to take care of their own family [12]. Hence, parents who marry their children off early “are not necessarily heartless parents but, rather, parents who are surviving under heartless conditions”, as some parents use child marriage as a strategy to break out of poverty [19].

### Negative effects of child marriage

There are many reasons why child marriage is perpetuated, which can be beneficial in many ways. However, empirical evidence suggests that on the balance, the same reasons that make child marriage beneficial are the same reasons that make it problematic with various negative socio-economic and health effects to girls, their children, families and their communities.

Evidence show elevated rates of suicidal thoughts or attempts among girls promised or requested in marriage and married girls compared to those not yet in the marriage process, suggesting that child marriage is a problem at the very onset even before sex and child bearing [20]. Child marriage is a form of violence against young girls as it increases their vulnerability to sexual, physical and psychological violence due to the unbalanced power dynamics within marriage [21, 22].

While child marriage is usually used to ensure that sex and child bearing occur within marriage, it effectively brings a girl’s childhood and adolescence to a premature end and imposes adult roles and responsibilities on young girls before they are physically, psychologically and emotionally prepared to handle them [23]. Sexual intercourse and child bearing among girls can lead to various health complications, however, the practice of child marriage worsens these health challenges. For instance, early sexual debut goes hand in hand with child marriage, which increases a girl’s health risks, because an adolescent’s vaginal mucosa is not yet fully matured, exposing them to increased risk of sexually infected diseases including HIV [24]. In 29 countries including Ghana, it was found that female adolescents were more vulnerable to HIV infection than older women. Women who marry young often tend to have much older husbands, in polygamous unions and are frequently junior wives which increases young girls’ probability of HIV infection [25].

Child marriage will most likely result in early child bearing resulting in serious health implications. The mean age at first birth of girls who marry early is approximately 2 years younger compared to women who marry as adults [21, 24]. Further, early pregnancy loss among girls age 15–19 has been found to be twice as high as that of other age groups in Ghana [26]. For instance, the 2014 GDHS reported that neonatal (42 deaths per 1000 live births), infant (62 deaths per 1000 live births), and under-5 mortality (84 per 1000 live births) were highest among children born to mothers less than 20 years compared to those aged 20 years and above [27]. In another study in Ghana, it was found that first-born children of women who married before age 18 had increased odds of mortality compared to first-borns of women who married after 18 years [21]. Thus, child marriage, exposes girls to exacerbated intergenerational health risks as they are exposed to various reproductive health challenges, children born to them have higher mortality rates and are more likely to be born prematurely [21]. Aside reproductive health challenges, child marriage has also been found to be associated with increased likelihood of difficulties with activities of daily living (including carrying a 10 kg load for 500 m; bend, squat or kneel; and walking a distance of 2 km) [21].

A common belief is that child marriage is a coping strategy for poverty, accords girls and parents status and honour. However, evidence also show that child marriage is a catalyst for poverty which undermines status and honour in societies. In sub-Saharan Africa including Ghana, it was found that early marriage negatively influences education as it reduces the probability of literacy and completing secondary school [28]. In Ghana, early marriage among girls has been found to be one of the important challenges facing effective enrolment and school attendance, which leads to school dropout [29]. In essence, it ends a girl’s opportunity to continue her education to acquire employable skills, which results in persistent poverty among girls and effectively undermines their status and honour as they are unable to meet their daily needs [12, 19, 30].

### Legal norms in relation to child marriage

Child marriage undermines the fundamental human rights of children and violates Article 16(2) of the Universal Declaration of Human Rights, which states that “Marriage shall be entered into only with the free and full consent of the intending spouses”. It also violates Article 16 of the Convention on the Elimination of all Forms of Discrimination Against Women (CEDAW) that women should have the same right as men to “freely choose a spouse and to enter into marriage only with their free and full consent”.

The 1998 Children’s Act of Ghana and the 1992 Constitution of Ghana define a child as a person below the age of 18. By age 18, young persons are expected to have developed enough intellectual, emotional and physical skills, and resources to fend for themselves as well as to successfully transition into adulthood. Until then they require care from adults, support, guidance and protection [31]. The 1998 Children’s Act of Ghana (Act 560), indicates that no person shall force a child: (1) (a) to be betrothed; (b) to be the subject of a dowry transaction; or (c) to be married; and (2) the minimum age of marriage of whatever kind shall be eighteen years (18 years).

In Ghana, there is commitment towards curbing child marriage. The Ministry of Gender, Children and Social Protection established a Child Marriage Unit in 2014 to promote and coordinate national initiatives aimed at ending child marriage in Ghana. In 2016, the unit in partnership with the United Nations Children’s Fund (UNICEF) and other key stakeholders developed a National Strategic Framework on Ending Child Marriage in Ghana. The framework is to ensure effective, well-structured and well-guided collaboration between state and non-state institutions [32].

Despite signing on to international resolutions, national laws, and efforts by various national and international organizations, child marriage in Ghana remains a phenomenon of concern with very limited empirical evidence to support program interventions to deal with the practice. The present study seeks to (a) identify the predictors of child marriage in the broader Ghanaian society and (b) explore in-depth the norms and practices surrounding child marriage as well as how the phenomenon could be addressed.

## Methods

The study employed a multiple-method approach to achieve its objectives. The study utilised quantitative data from the women’s file of the 2014 Ghana Demographic and Health Survey (GDHS) to examine the predictors of child marriage. This was complemented with qualitative data collected in purposively selected districts and communities in United Nations Population Fund (UNFPA) country program support regions (Central, Northern and Greater Accra) in 2016 to examine norms and practices surrounding child marriage. These were regions with high prevalence of teenage pregnancy (Central, 21.3%) and child marriage (Northern, 35.8%) [27]. The Central region is in the southern part of Ghana along the coast. The people in the region are generally of the Akan ethnic group and matrilineal. It is bordered by Ashanti and Eastern regions to the north, Western region to the west, Greater Accra region to the east and the Gulf of Guinea to the south. The Northern region is in the northern part of the country and the people are predominantly of the Mole-Dagbani ethnic group and patrilineal. The region is bordered on the north by the Upper West and Upper East region, on the east by Togo, on the south by Brong Ahafo and Volta regions, and on the west by Côte d’Ivoire.

### Quantitative procedure

The GDHS data is a nationally representative survey that was first conducted in 1988 and has since been conducted roughly every 5 years. The GDHS collects data from women aged 15–49 and men 15–59 years on various topics including socio-demographic characteristics and age at first marriage [27].

### Variables

#### Dependent variable

The dependent variable for this study is child marriage. The child marriage variable is dichotomous, where 1 indicates an individual woman first married/cohabited before age 18 and 0 otherwise. In this paper, the analysis of child marriage is restricted to women aged 20–24 years to ensure that no respondent was still at risk for marriage during adolescence [22, 23]. This resulted in a sample size of 1571 (weighted sample size = 1613).

#### Independent variables

The independent variables considered in this study were ever attended school (yes, no), religion (Christian, Muslim, Traditional/Spiritualist, No religion), ethnicity (Akan, Ga/Dangme, Ewe, Mole-Dagbani, Gurma, Other), region (Greater Accra, Western, Central, Volta, Eastern, Ashanti, Brong Ahafo, Northern, Upper East and Upper West), residence (urban, rural) and wealth quintile (lowest, second, middle, fourth, highest).

#### Data analysis

Frequencies were used to describe the characteristics of respondents in the sample. The logit model was used at two levels, first, to examine the bivariate relationships between each of the independent variables and the dependent variable without accounting for other factors. Second, to examine the net effects of each of the independent variables on the dependent variable controlling for other variables. The logit regression model finds the best fitting model to describe the relationship between the dichotomous variable of interest and a set of independent variables [33]. The logit coefficients do not have an intuitive interpretation because they represent effects of the log of the odds. For easier interpretation, the log odds, are converted to odds ratios by exponentiation [33]. Only the odds ratios are presented for the logit regression models in this study. The basic logit regression model takes the form:

3.1 $$ \ln\ \left(\frac{\mathrm{pi}}{\left[1-\mathrm{pi}\right]}\right)=\mathrm{bo}+\mathrm{biXi} $$

Where *pi* is the estimated probability of a particular event occurring to an individual with a given set of characteristics, *bo* is the intercept, and *bi* represents the slope coefficients for a set of explanatory variables *Xi*.

The quantitative analysis was conducted using STATA (version 13). To correct for non-response and ensure representativeness across the country, the data was weighted taking into account the Demographic and Health Survey (DHS) complex survey design using the ‘svyset’ commands [34]. The *svy* prefix command *subpop* option was used to restrict the sample to women aged 20–24 years [34].

### Qualitative procedure

The qualitative component of this study involved focus group discussions (FGDs) and Key Informant Interviews (KIIs) with stakeholders (adolescent girls, young women, parents, community leaders and those working directly or indirectly on issues affecting young people aged 10–24 years) on child marriage. The discussions covered norms and practices surrounding child marriage as well as how the phenomenon can be addressed. The KIIs and FGDs were conducted from June to August 2016. Participants for the FGDs and KIIs were recruited through key contacts in various organizations, Microfin, World Education Ghana and Ghana Health Service in the Central region and NORSAAC, Ghana Health Service and ActionAid in Northern region. The purpose of the study, the target population, as well as period of the study, and other details including mobilization of participants, logistics, transportation and community entry were discussed with the key contacts. Once feasibility was established, the key contacts identified community volunteers to mobilize eligible participants. The volunteers and key contacts sought audience with the traditional and local authorities approximately 10 days prior to data collection to inform them of the purpose of the study, target groups and key persons as well as seek their permission to conduct the research in their respective communities.

The qualitative data was used to build on statistical results by adding meaning, context and depth. Semi-structured interview guides were used for the FGDs (Additional file 1: Appendix A) and KIIs (Additional file 2: Appendix B), with a set of questions, however, questions that were not included in the guide were also asked as the interviewers probed further on things said by participants.

#### Study sites

The focus group discussions (FGDs) targeted the northern and southern sectors of Ghana. These are regions with high prevalence of teenage pregnancy (Central: 21.3%) and child marriage (Northern: 35.8%) [27].

#### Focus group discussions (FGDs)

Focus group discussions were conducted in three communities in the Central region (Asubo-Awutu, Obidan and Dosii-Central) and in four communities in the Northern region (Zabzugu, Sabare, Tasundo and Kukpaligu). In each region, 10 focus group discussions were conducted (20 FGDs in total). Each focus group discussion had a maximum of 10 participants. The FGDs were conducted among the following subgroups: 12–17-year olds who were married, 18–24-year olds (who got married before the age of 18), unmarried 12–17-year olds (at risk of child marriage) and unmarried 18–24-year olds. Married 12–24-year olds were asked questions specifically about their lived experiences within marriage, while unmarried 12–24-year olds were asked about their motivations to delay marriage. FGDs were also held among parents/guardian, grandparents, and other adult community members. Male and female parent FGDs were conducted in Kukpaligu in the Zabzugu-Tatale district at the request of the community members, indicating that women would be reluctant to talk in a mixed gender setting.

#### Key informant interviews (KIIs)

Were conducted with focal persons/key informants/key stakeholders in government institutions (Ghana Education Service, Social Welfare, Ghana Health Service, Ghana Police Service with special attention on the Domestic Violence and Victims Support Unit, Parliament), and non-governmental (World Vision, Hope for Future Generation, Compassion International), as well as at the community level (Christian and Muslim leaders, Chiefs, other Community leaders and representatives, head teachers). Thirty (30) KIIs were conducted in the Central and Northern regions to get regional perspectives on child marriage, and in Greater Accra region with national representatives to get a national view on issues surrounding child marriage.

#### Data analysis

Research assistants transcribed (some with the help of a translator) the audio-recorded interviews and discussions verbatim into English. Codebooks modelled initially around topics of the interview guides were developed. Through the iterative process of coding and analysis, codes were added to the codebook. The transcripts were coded manually, guided by open and axial coding. To ensure inter-coder reliability, transcripts were analysed by a team of 5 persons (research assistants and principal investigator). The initial codes generated were then grouped into preliminary categories of themes. Through reading, re-reading and constant comparison, the preliminary categories of  themes were categorized into themes and sub-themes.

## Results

Descriptive results in Table 1 show that about one in five (20.68%) of the women in the sample first married before age 18 (mean age at first marriage among girls aged 20–24 years = 17.7 years; std. dev. = 2.6; minimum age at first marriage = 10 years (0.18%, 3 women) and maximum age at first marriage = 24 years). A little more than one out of ten (11.92%) had never attended school. Four fifth (80.33%) of the women were Christians, 15% were Muslim, about 1 % were Traditionalist and 3 % had no religious affiliation. Half (49.66%) of the respondents belonged to the Akan ethnic group. The highest proportion of the respondents resided in Greater Accra region (20.74%) and the least in Upper West region (2.38%). A higher percentage of the respondents were in Urban areas (53.28%) and 26% of the women in the sample were in the fourth wealth quintile category.

**Table 1:** Characteristics of the sample, women age 20–24

Variable	Number	Percent
Age at first marriage		
< 18 years	334	20.68
18 years and above	1279	79.32
Ever attended school		
Yes	1421	88.08
No	192	11.92
Religion		
Christian	1296	80.33
Muslim	247	15.31
Traditionalist	21	1.31
No religion	49	3.05
Ethnicity		
Akan	801	49.66
Ga/Dangme	119	7.37
Ewe	218	13.50
Mole-Dagbani	247	15.29
Gurma	101	6.27
Other	127	7.90
Region		
Western	189	11.74
Central	151	9.36
Greater Accra	335	20.74
Volta	118	7.31
Eastern	165	10.24
Ashanti	293	18.16
Brong Ahafo	131	8.12
Northern	137	8.49
Upper East	56	3.46
Upper West	38	2.38
Residence		
Urban	859	53.28
Rural	754	46.72
Wealth quintile^a^		
Lowest	229	14.17
Second	260	16.10
Middle	387	23.99
Fourth	413	25.58
Highest	325	20.16
Total (weighted)	1613	100.00

Source of data: 2014 GDHS

^a^this may not sum up to 1613 due to rounding offs

Table 2 shows bivariate logistic regression results of the relationship between child marriage and each of the background variables. The results reveal a significant relationship between education and child marriage with women who had never attended school being more likely to marry as children. Except for Traditionalist/Spiritualist, Muslim women and women with no religion were significantly more likely to marry as children. With respect to ethnicity, women belonging to the Mole-Dagbani, Gurma, Other ethnic groups were significantly more likely to marry as children compared to Akan women. Women in Eastern, Northern, Upper East and Upper West were more likely to marry early compared to their counterparts in Greater Accra. Women in rural areas compared to women in urban areas were significantly more likely to marry as children. In addition, women in the middle, fourth and highest wealth quintiles were significantly less likely to marry early compared to those in the lowest quintile.

**Table 2:** Bivariate logistic regression predicting child marriage, women age 20–24

Variable	OR	95% CI	
Ever attended school {Yes}			
No	4.07***	[2.86,	5.79]
Religion {Christian}			
Muslim	1.86***	[1.28,	2.71]
Traditionalist/Spiritualist	2.59	[0.78,	8.60]
No religion	2.96***	[1.41,	6.22]
Ethnicity {Akan}			
Ga/Dangme	0.72	[0.37,	1.39]
Ewe	0.69	[0.43,	1.10]
Mole-Dagbani	1.63*	[1.11,	2.38]
Gurma	2.02***	[1.27,	3.21]
Other	1.64*	[1.04,	2.59]
Region {Greater Accra}			
Western	1.22	[0.68,	2.19]
Central	1.16	[0.67,	2.01]
Volta	1.01	[0.53,	1.94]
Eastern	1.61+	[0.98,	2.67]
Ashanti	0.73	[0.41,	1.30]
Brong Ahafo	1.32	[0.76,	2.29]
Northern	2.66***	[1.64,	4.30]
Upper East	1.91+	[0.95,	3.88]
Upper West	2.30+	[0.93,	5.68]
Residence {Urban}			
Rural	1.97***	[1.46,	2.65]
Wealth quintile {Lowest}			
Second	0.71	[0.46,	1.09]
Middle	0.44***	[0.30,	0.65]
Fourth	0.28***	[0.17,	0.46]
Highest	0.25***	[0.15,	0.43]

Exponentiated coefficients; 95% confidence intervals in brackets []; Reference categories in parenthesis {}; + *p* < .1, * *p* < .05, *** *p* < .001

Source of data: 2014 GDHS

### Drivers of child marriage

Table 3 shows results of binary logistic regression model predicting the net effects of each of the independent variables on child marriage controlling for other variables. The results show that the odds of a woman marrying as a child was 3 times more likely for those who had never attended school compared to their counterparts who had ever attended school.

**Table 3:** Results of multivariate logistic regression model predicting child marriage, women age 20–24

Variable	OR	CI
Ever attended school {Yes}		
No	3.01***	[1.90, 4.78]
Religion {Christian}		
Muslim	1.40	[0.86, 2.29]
Traditionalist/Spiritualist	1.34	[0.38, 4.67]
No religion	2.12+	[0.97, 4.63]
Ethnicity {Akan}		
Ga/Dangme	0.50+	[0.24, 1.07]
Ewe	0.46*	[0.24, 0.87]
Mole-Dagbani	0.82	[0.47, 1.43]
Gurma	0.64	[0.29, 1.42]
Other	0.94	[0.55, 1.63]
Region {Greater Accra}		
Western	0.65	[0.34, 1.23]
Central	0.67	[0.37, 1.22]
Volta	0.83	[0.33, 2.10]
Eastern	1.00	[0.59, 1.68]
Ashanti	0.50*	[0.28, 0.90]
Brong Ahafo	0.52*	[0.28, 0.96]
Northern	0.49+	[0.24, 0.98]
Upper East	0.50+	[0.22, 1.12]
Upper West	0.66	[0.28, 1.58]
Residence {Urban}		
Rural	1.25	[0.86, 1.82]
Wealth quintile {Lowest}		
Second	0.82	[0.45, 1.51]
Middle	0.59+	[0.33, 1.03]
Fourth	0.37**	[0.20, 0.71]
Highest	0.32**	[0.15, 0.69]
Total (Weighted)	1613	
Linktest		
Hat	0.01	
Hatsq	0.40	

Exponentiated coefficients; 95% confidence intervals in brackets []; Reference categories in parenthesis {}; + *p* < .1, * *p* < .05, ** *p* < .01, *** *p* < .001

Source of data: 2014 GDHS

In the qualitative component of this study, it was also found that education was an important reason for delaying marriage. Across the two study areas, when adolescent girls and young women were asked about their plans and reasons for delaying marriage, they often mentioned their educational goals as the reason for not marrying early. Adolescent girls indicated that education was the foundation of their life aspirations, recognizing that early marriage truncates educational achievements:

Yes, I planned for that. When you get married before 18 years you can’t further your education again. – FGD 18-24 Unmarried, Zabzugu

My family influenced my delay in marriage because I was always advised to further my education and be a better person before getting married. – FGD 12-17 Unmarried, Sabare

Parents also acknowledged how early marriage could derail educational achievements:

If she wants to further her education, she will say she will not marry. Unless she finishes her school before she will marry. – FGD Male Parents, Kukpaligu

When other variables are controlled for, the relationship between religious affiliation and child marriage becomes very weak. Only women with no religious affiliation were more likely to marry as children (OR = 2.12, significant at *p* < 0.1) compared to Christian women. Compared to Akan women, Ga/Dangme and Ewe women were less likely to marry as children. In comparison with Akan women, the odds of Ga/Dangme women marrying as children were 0.50 times less likely (significant at *p* < 0.1) and Ewe women were 0.46 times less likely.

Contrary to the bivariate results, the odds of women in Ashanti region marrying as children were 50% lower, Brong Ahafo was 48% lower, Northern 51% (significant at *p* < 0.1) lower and Upper East 50% (significant at *p* < 0.1) lower compared to their counterparts in Greater Accra region. There was no significant variation between women in rural and urban areas with respect to child marriage once other variables were accounted for. While women in the second wealth quintile were not significantly different from those in the lowest wealth quintile about child marriage, women in the middle, fourth and highest wealth quintiles were significantly less likely to marry as children compared to women in the lowest wealth quintile. The odds of young women in the middle, fourth and highest wealth quintile compared to those in lowest wealth quintile marrying as children were 0.59 (significant at *p* < 0.1), 0.37 and 0.32 times less likely (Table 3).

Consistent with the finding in the quantitative analysis, where women in the highest wealth quintile were less likely to marry as children compared to those in the lowest wealth quintile; it was found in the qualitative data that, poverty was a driver of child marriage in both regional settings. Adolescent girls and young women indicated that poverty was one of the main drivers of child marriage and this was common in both research settings:

When I ask my father for money, he says he doesn’t have, so that is why I got married early. – FGD 12-17 Married, Sabare

Sometimes, because of poverty some people give their children out. Your father may be in need and may ask for help from a rich man. After the man renders help to your father, your father will say let me pay this person back for the kind gesture he has shown me by giving you in marriage to that man. – FGD 18-24 Married, Kukpaligu

The reason why our females marry early is because some of our parents do not have, so if the man will be able to cater for you then it means that you should understand him. If my mother doesn’t have and I have somebody who can cater for me, I will understand him, for the pressure on my mother to be relieved. So that is why we marry so early. – FGD 18-24 Married, Obidan

Some parents don’t have, so they are unable to meet the needs of their children. And the children “by force” engage in relationships and it will result in pregnancy and she will end up entering marriage. – FGD 18-24 Unmarried, Dosii

The key informant interviews also revealed that poverty was one of the important drivers of child marriage. Some of the key informants indicated that some parents allow their girls to marry early to get something in return from the man:

I will say it is poverty, because parents always give the excuse that they are poor because of lack of employment in the system. They will say they do not have money to take care of the child so by the time they think you are of age they should just give you out for marriage; they will get something in return from that man. So, at the end of the day it is poverty. – KII, Social Welfare, Cape Coast

Well, I will say maybe poverty. One, like I said religious beliefs and traditional setup is also the cause of it because when there is poverty at home, some parents do not look at the consequences. Some even lure the children into it so that they get monies from their in-laws. Thus, poverty is one of the main issues that drive child marriage. – KII, Domestic Violence and Victim Support Unit (DOVVSU), Accra

Adolescent girls and young women described how some parents were either aware of or encouraged their relationships borne out of a lack of money/wealth at the family level:

Some of the girls’ parents don’t have money, so when she meets a man who promises to help her in school, she will go and tell her mother that this man says he will help her in school. Then the mum then agrees to it and from there she will be courting with the guy and suddenly she gets pregnant. – FGD 12-17 Married, Awutu Asubo

On the other hand, particularly in the Central region, some parents acknowledged that their children engaged in transactional relationships because of family hardships, which leads to marriage:

Some are experiencing hardship, so when the girl goes to meet someone who is wealthy, the mother forces her to marry that person. It is not the time for her to marry, but because of hardship and the wealth of the man, she will be forced to marry him so that he can take care of her. – FGD Parents, Assin Dosii

Despite the family’s economic circumstances, not all adolescents held their parents responsible for entering early marriage. Some girls from both regions felt that since they did not have money to go to school the best alternative was to marry early:

As I am schooling, I don’t have anybody taking care of me, my parents are poor, but they did not force me to the man. Because my parents are poor, I have nothing to offer myself. That is why I got married early. – FGD 12-17 Married, Sabare

I went into marriage because there was no money. If I look back, there is no one, that is why I had to force <<do what it takes>> to get married. – FGD 18-24 Married, Obidan

It is hardship. In my case, my father did not pay my fees when I was about to complete, and the man promised to pay but the registration was over. But he helped me learn a trade and I got pregnant, so he brought me here that is the reason why I got married so early. But it was not as if somebody forced me. – FGD 18-24 Married, Awutu Asubo

Establishing causal relationship as to whether pregnancies occurred before marriage or that pregnancy led to early marriage was beyond the scope of this study. However, the focus group discussions in the two qualitative study settings revealed that teenage pregnancy was one of the main drivers of child marriage:

Yes, teenage pregnancy can lead to early marriage because we Muslims when you get pregnant you cannot live in your parents’ house; you should move to your husband’s house. We do not wish to go into early marriage, but immediately we get pregnant, our parents say that as far as they are concerned, we should move in with the men. – FGD 12-17 Married, Sabare

I went in for a boyfriend and whatever I asked him, he give to me. I got pregnant, I stopped school, and I am now living with him. So, it is pregnancy that led me into marriage. – FGD 12-17 Married, Obidan

What I also know is that some of the girls are in primary or junior secondary school (JSS) and before you realize the person is pregnant. So, this can make the person marry early. – FGD 18-24 Married, Kukpaligu

Parents offered their perspectives on how and why teenage pregnancy usually leads to child marriage. Some parents indicated that when a child falls pregnant, they will let the man responsible for the pregnancy marry the girl even if she does not want to enter early marriage:

When she is underage and conceives, they will give her to marry that boy. You the father will be thinking that she is not ready, but you will see that she is pregnant, so you should give her out for marriage. – FGD Male Parents, Kukpaligu

For us the mothers, we think that if you have a child, the child should live with you until she is ready for marriage. But before you realize the girls will bring pregnancy to you. Hence, the child would be forced to go to <<marry>> whoever impregnated her. – FGD Female Parents, Kukpaligu

In the key informant interviews, it also came out that the practice of parents forcing girls to marry men responsible for their pregnancy was a common phenomenon in their communities:

Yes, for some people, if someone impregnates your daughter, they will just give her to that boy to marry. Yes, to marry. – KII, Opinion Leader-Kukpaligu

Yeah, some of them their parents look out for those who put them in the family way <<pregnant>> and then they see their parents and they marry them. – KII, Opinion Leader-Zabzugu

Yes, I’ll say yes. Especially in this district, what the people in the district are doing now is that; when you get a teenager pregnant, they ask you to come out and pay the bride price to legalize whatever you have done before you can even name the child. So, that is really causing more child marriages than before. – KII, Ghana Health Service-Zabzugu

[Pauses briefly to think about response to the factors that influence child marriage] Teenage pregnancy too can be one of them. – KII, Teacher at Assin Dosii

Some adolescents and young women recognized their own role in getting married or being in a union because of teenage pregnancy. They indicated that in some cases, parents could not be blamed for early marriage, as it is the girls who fall pregnant. Indeed, some of the girls insisted on marrying the men who made them pregnant:

What I know is that sometimes it is not the will of the parents that the children marry early. It is the fault of the children themselves. The person will be in school and before you realize she is pregnant. And when she is pregnant, she has to go into marriage. It is not the making of the father and the mother. – FGD 18-24 Married, Kukpaligu

When I was in school, a guy proposed to me and I accepted. After some time in the relationship, I got pregnant. When that happened, my parents were unhappy about it and did not agree for me to marry the man. I refused to listen to my parents and married the man. – FGD, 12-17 Married, Awutu Asubo

### Socio-cultural drivers of child marriage

Reasons for child marriage vary from one society to the other. The data revealed that socio-cultural factors such as betrothal and exchange of girls for marriage were common in less developed settings in the Northern region. The betrothal of young girls, which was common in the Northern region, was also mentioned as a cultural practice that drives child marriage within some communities (Zabzugu-Tatale):

For example, just like I’m having this baby, my husband’s mother will call his son and tell him when his child is grown, she would come for her as a wife for a particular man for marriage. – FGD 18-24 Married, Kukpaligu

What I also know is that while the children are young their parent will show them their husband. So, because of that, the person will be eager to enter it because she has a husband already. – FGD 18-24 Married, Kukpaligu

Aside from betrothal of young girls, in the focus group discussions, among the *Konkombas* of the Northern region, there was the cultural practice of exchange of girls for marriage by families, which was a main driver of child marriage in that area:

Most of us are exchanged, the person will go and bring her sister to your brother and your brother too will give you to that man. Because of that, we are marrying early. You are small, and your brother doesn’t have a wife, he will use you to exchange like that. – FGD 12-24 Married, Tasundo

Your uncles will use you for exchange, so they will like to send you quick so that they get theirs quick. – FGD Female Parents, Kukpaligu

The key informants in the Zabzugu-Tatale district also mentioned the culture of exchange of girls among the *Konkombas* as one of the drivers of child marriage in the area:

The main cause is their culture and they don’t want to leave that practice. They still exchange girls and when you marry, and you don’t have a girl to give the other family, they will take your wife. So, they are compelled to give them. When you give them and she is a small girl, they will take her but if you don’t have, they will take your wife. So sometimes they will take them out of school and exchange. – KII, Religious Leader-Kukpaligu

In describing these de-facto practices, the lack of consent of girls and the forced nature of these marriages were very apparent. Girls had no option than to marry the man their family members betrothed them to. In some cases, even the mothers of the girls do not have a say when the girls are being exchanged for marriage. Some of the participants indicated:

When you are young, your father will give you out for marriage so whether you like it or not, you’ll have to go. And when it happens like that, you can’t do anything than to agree. – FGD 18-24 Married, Kukpaligu

You the mother will be sitting there, and the uncle of the child will come and just tell you that they are taking your daughter to this community for a wife. If you say no, they will beat you and the girl and force the girl to the place. – FGD Female Parents, Kukpaligu

Bride wealth was another dimension related to the persistence of child marriage. It is a cultural phenomenon in most Ghanaian societies. In the focus group discussions, girls believed that because bride wealth is cheap, men find it easy to pay and ask for the hand of young girls for marriage. Some participants therefore felt that an increase in bride wealth could serve as a deterrent to delay age at which girls get married:

They should make the wedding things expensive. If it is expensive, it is like if the man goes and he has not got money, he can wait. Maybe when the girl is 17, he will wait till the girl is 20 before, he will have money then, to buy the things. – FGD 12-17 Unmarried, Dosii

I think if the bride price is increased, it will make the men not able to afford it, so they will not be able to pay, and this will make us wait till we get to the right age of marriage. Because if the bride price is low, the moment the man pays it, he insists you get married as early as possible, therefore increasing the bride price will make early marriage stop. – FGD 12-17 Unmarried, Sabare

Pressure to get married at an early age can come from various significant others, namely family, society, peers and self. Some parents/family encourage or pressure their daughters to get married early by always comparing them to their peers who are already married:

From parents. They see your colleagues marry, then they tell you that, ‘you have seen your colleagues marrying and you are there, so you too hurry up and marry’. Thus, the pressure is from the parents. – FGD 18-24 Unmarried, Zabzugu

Sometimes the family. You know, you live with your parents, you live with your family members and most of the elders in the family will put pressure on you to marry. They will say look at this person, maybe she is your cousin or family member, she has married and maybe you are older than her and she is married, and you are still there. Through that you can even force someone to marry you. – FGD 18-24 Unmarried, Zabzugu

In the focus group discussions, it was found that marriage is cherished as it is in most African societies and unmarried young girls are usually teased or mocked because they are not married. Hence, some girls would want to get married early just to conform to the status quo:

Some want early marriage because of mockery. Sometimes, those who get married tend to make mockery of those who are not married, they ask them to accord them the respect simply because they married early. – FGD 12-17 Unmarried, Sabare

Some young girls enter early marriage because their colleagues are married or when they see their friends doing very well in marriage, they also want to get married. This was also noted in the KIIs. In other cases, young girls might go into relationships early for economic gains:

“When you see that your friends you walk with are getting married, you also want to get married. That is why we get married early.” – FGD 18–24 Married, Kukpaligu

It is not because of anything that we girls are in a hurry to get married, it is because of peer pressure. When we see others like us being treated very well in their marriage, we get attracted and try to marry to be treated well <<laughs>> – FGD 12-17 Unmarried, Sabare

I also think it is bad influence that causes it, we listen to what our friends say. Sometimes a friend may have fancy clothes and you may be envious so that friend will tell you I slept with a man to get them, so you can also get a man who will look after you so that you can also get the clothes I have. She will also listen to her friend and go in for a man as her parents cannot provide her with those clothes.” – FGD 18–24 Unmarried, Obidan

Sometimes it is from the peer group. Peer group influences. – KII, Opinion Leader-Kukpaligu

In the FGDs as well as from the KIIs, it was revealed that some girls decide to marry early, indicating that it was their own will or out of curiosity and in some cases, out of stubbornness (not listening to their parents’ advice):

Our parents cannot force us to go and marry the men, but we did ourselves because of our own curiosity. Even though they advised us against it, we refused. That is why we are suffering like this. – FGD 12-17 Unmarried, Sabare

Nobody forced me to get married early, I forced myself to marry because I was schooling, and nobody was taking care of me that was why I got married. – FGD 12-17 Unmarried, Sabare

It was because of my stubbornness; my parents did whatever they could to cater for me. It was as result of my stubbornness and peer pressure that has landed me in such marriage. – FGD 18-24 Married, Obidan

Yes, stubbornness because regardless of what you say the children do not listen or take it when mothers talk, they just don’t listen. Some also develop early, for instance at thirteen years, they menstruate so by fourteen when they go for a man, they get pregnant. When they get pregnant too, they will have a baby . . . they don’t listen to their parents oh! If we try to correct them, we are not able to do so at all. – KII, Queen Mother, Central Region]

### Ending child marriage

To help curb the practice of child marriage in Ghana, participants in the FGDs highlighted the role of the police. Adding that instead of giving a girl out for marriage because of pregnancy the man responsible should rather be arrested:

Taking the case to the police station will make it stop. – FGD 12-17 Married, Sabare

To stop early marriage, when a girl gets pregnant whilst in school, the man responsible should be arrested and this will make it stop. **–** FGD, 12-17 Unmarried, Sabare

Participants also spoke fervently about the authority and role of the chiefs in their communities in ending the practice. Participants indicated that the chiefs should be more vocal against child marriage and that women should make it a point to report their husbands to chiefs when they are going to give their girls out for marriage:

If your husband is forcing your children to marry early, you should report your husband to the chief. – FGD Female Parents, Kukpaligu

The chief in this village can make this child marriage stop because if he can open his mouth and talk about it, it will stop. But if an elder says it, they will not believe him unless the chief himself says it. If the chief decrees it himself, they will fear him and stop. – FGD 18-24 Unmarried, Obidan

Participants went further to explain the need for community-based laws and policies that should be established specifically by the chief of the community and elders, which they believe, will help curb child marriage:

The chief of this community and his elders can impose their laws and it will stop. **–** FGD 12-17 Married, Zabzugu

The chief can pass the law and it will work because everybody wants it to stop. – FGD Female Parents, Kukpaligu

Madam, they [elders] should bring a rule that tells parents to make sure their children sleep early so if they pay attention to the children and take care of them it can make all the child marriages and teenage pregnancies stop. – FGD 12-17 Unmarried, Obidan

All that can be done is that elders and opinion leaders must make laws that any man who impregnates a girl who is in school or under age should be arrested and this will put some fear in them. – FGD 12-17 Unmarried, Sabare

Key informants also indicated that chiefs have a key role to play in curbing child marriage. They suggested that chiefs should establish laws on child marriage and ensure offenders are punished:

For the chiefs, they can even establish some laws within the community that whenever you do this these are the punishments that you are going to face, and I think no one is ready for a punishment. Through that I think we can reduce the child marriage. – KII, Teacher, Central Region

Yeah, the police we have partnered with the UNFPA sensitizing the villagers on such issues or activities. They should not involve themselves in it. – KII, Police Officer-Zabzugu

The education of girls was regarded as both a protective factor against early marriage and a means to curbing or ending the practice. Participants in the FGDs pointed out that when a girl is enrolled in school, she cannot be given out for marriage easily. This view was shared in both the parents and girls focus group discussions:

When you don’t want to marry early, you go to school direct. When you enter school, they won’t give you like that. – FGD 12-24 Married, Tasundo

No matter how high the bride price, so far, the husband is willing to marry the child they will still pay. The only thing is we will bring our heads together and educate the girl on certain things and advise the girl that if she goes to school it will be better for her in future than if she gets into marriage. – FGD Female Parents, Kukpaligu

I think the only way to stop early marriage is through education. If the level of educating the girl child is intensified, early marriage will stop. – FGD 12-17 Unmarried, Sabare

Teenage pregnancy and marriage won’t happen if you are in school because you want to do something good. Since our friends have done it and it looks nice, we should also learn so that all these things can help us. – FGD 12-17 Married, Obidan

Key informants also echoed education as a way of ending child marriage among girls, indicating that education will not only help delay marriage but also empower the girls:

We must make sure that girls’ education is enforced at all levels and the welfare system. – KII, Head of NGO, Greater Accra

We are trying to look at it from every angle, it is education. That is why there are a lot of sayings about girl child education. In our religion, it is said that when you educate a girl child, you have much more blessings and that also go with the philosophical sayings of the great men in our society like Aggrey If you educate a girl, you educate a whole nation. – KII, Muslim leader, Central region

In addition, some of the key informants indicated that one of the ways to end child marriage was through awareness creation and advocacy on the consequences of child marriage:

Absolutely yes, we have been embarking on sensitizations in the communities, because it is the parents who should support the children to go school. A child cannot take herself to school even if she can take herself to school there should be support, financially, morally, everything. So, we sensitize the parents and we sensitize the girls. The girls’ education unit especially sends me to go out to the schools with my colleagues and then we talk to the girls, we sensitize and inspire them to go high in education and we tell them the prospect or the benefit of education. So, we’ve been doing a lot of activities, we have *Ahomeka*, a local radio station, we’ve been going there to educate the public on the importance of education.” – KII, Girl-child Education Officer, Central Region

Some of the key informants also indicated that, there are laws in Ghana to curb child marriage, but the challenge was with the enforcement of the laws:

The policies are there, because our law is clear on child marriage. Hence, if people marry a child at the age of fifteen, it is against our laws, but there is no punishment, you understand. – KII, Head of NGO, Greater Accra

In my opinion, the laws on child marriage are weak. When it comes to child marriage issues, it is like there are no sanctions. I have never witnessed a parent being sanctioned for giving out his or her child for early marriage. I am yet to see that. So, I feel stiffer punishment should be there for people who do that. So that it would scare the others from doing it. – KII, Staff of Ghana Health Service, Zabzugu

## Discussion

This study sought to identify the predictors of child marriage in Ghana and explored norms and practices surrounding child marriage as well as how the phenomenon can be addressed. It is worth noting that girls aged 12–15 years compared to girls aged 16–17 years who are sexually active, married or mothers raise different issues. However, this discussion is beyond the scope the present study. From the quantitative data, one in five (20.68%) young girls aged 20–24 years married as children, a reduction from 24.58% in the 2008 GDHS data. At this rate of decline, Ghana will most likely not meet the sustainable Development Goal 5, Target 5.3 which seeks to eliminate all harmful practices, such as child, early and forced marriage and female genital mutilations by 2030 [35].

This is considerably lower compared to findings from other developing country contexts. For instance, in five Indian states it was found that 63% of women aged 20–24 years were found to be married before age 18 [36]. This can plausibly be explained by some of the key activities implemented by the Child Marriage Unit which include the establishment of an Advisory Committee composed of influential individuals to tackle child marriage; formation of a network of stakeholders for experience sharing on best practices, lessons learnt and guidance on what works and what strategies do not work; launch of the Ending Child Marriage Campaign in Ghana in 2016; public sensitization through the use of popular Ghanaian personalities and the mass media; engagement with the youth to get their ideas on how to end child marriage; and engagement with the African Union and other actors at the continental level to share and learn from other African countries their efforts to end child marriage [32]. This could also be the result of significant increase in girl-child education in Ghana resulting in the decline of the incidence of early marriage (also see [7]). Young girls who had never been to school were more likely to marry as children compared to their counterparts who have ever been to school. This finding is in tandem with similar studies in Ghana and other sub-Saharan African countries [22, 37]. This underscores the importance of education as a preventive measure to child marriage.

Contrary to other studies in Ghana and other developing countries [6, 37], the results of the present study revealed that while young girls with no religious affiliation were marginally more likely to marry as children compared to their Christian counterparts, young Muslim girls were not different from young Christian girls. The differences in the findings could be attributed to the age range of women in the samples considered in the respective studies or context. Plausibly, the influence of religion is waning in modern Ghanaian societies as demonstrated in other studies (e.g. [38]). There were elements of cultural influence on child marriage as ethnicity and low bride wealth were found to be related to child marriage. This can be attributed to the high value placed on girl-child virginity as a source of honour to the family and higher bride wealth [13].

The results indicated that young girls in the Ashanti, Brong Ahafo, Northern and Upper East regions were less likely to marry as children compared to their counterparts in Greater Accra region. The reversal of these results in the relationship between region and child marriage in the bivariate and multivariate analysis can be explained by accounting for other factors in the multivariate model. The results especially in the Brong Ahafo, Northern, Upper East regions, can plausibly be explained by the continuous efforts by development partners such as UNFPA in those regions to curb child marriage. In addition, perhaps the traditional marriage process is being bypassed in the Greater Accra region attributable to the level of modernization or development in the region. In such modernized or developed settings, parents and families are less involved in individual marital preferences or strategies than they were in previous generations or in less developed or modernised context. Hence young girls can go into marriage early or live with men (cohabit) as if they are married without repercussions (also see [7]).

The influence of modernization or development was also manifested in the qualitative findings with the existence of socio-cultural practices such as betrothal and exchange of girls for marriage as marital strategies, which was common in less developed settings in contrast to modernised settings (Zabzugu-Tatale district in the Northern region). In some communities in the district, young girls were betrothed as early as when they were born and once they grow up they had no choice but to marry the men they were betrothed to. The results showed that these were cultural practices the communities were reluctant to let go despite their negative consequences.

Household economic status appeared to be significantly related to child marriage. The results revealed that young girls in the middle, fourth and highest wealth quintiles were significantly less likely to marry as children compared to their counterparts in the lowest wealth quintile. This finding corroborated with the findings from the qualitative data suggesting that poverty was also a crucial factor influencing child marriage. Parents who could not take care of the needs of their young girls either encouraged or forced them into early marriage [5, 12, 39]. In other cases, it was the desires and wants of young girls that led them into early marriage. The data showed that what usually led to teenage pregnancy was poverty, where the family of young girls were unable to take care of their needs in school and their social life. Once a girl got pregnant, she was forced to marry the man responsible for the pregnancy. These issues suggest that child marriage is used as an economic strategy for upward social mobility by girls and their parents in some instances. However, some girls acknowledged that it was not only poverty that led them to child marriage but asserted that it was sometimes their own stubbornness, inquisitiveness or materialistic desires that made them marry early.

Pressure from significant others also appeared to influence child marriage. Parents and other members of the society usually pressurized young girls into child marriage by comparing them to their peers who were married and sometimes appear to be doing well in their marital homes. The results further showed that some young girls enter child marriage simply because their peers were married, not knowing the negative effects of child marriage as some of the young girls lamented in the FGDs. From the results, there are strong similarities in terms of the drivers of child marriage in the two regional settings, even though they differ in several aspects such as language, lineage system and socio-economic development. Perhaps, this reiterates the widespread nature of the phenomenon though the context may differ.

Participants were asked how child marriage could be ended and they proffered various solutions. Participants in the qualitative interviews indicated that advocacy would be useful in curbing the practice. Hence tailored advocacy programs for adolescent girls should be developed. These programs should focus on educating communities and raising awareness on the consequences of child marriage. Additionally, the programs should be designed to equip community members to challenge insidious socio-cultural practices such as betrothal and exchange of girls for marriage. This could be done through opinion leaders such as chiefs to influence public opinion. Conscious efforts should be made by duty bearers to initiate a discourse for a specific policy on child marriage since the Children’s Act of Ghana does not comprehensively address the issue of child marriage.

The education of girls was regarded as a protective factor against early marriage. Participants in the focus group discussions indicated that when a girl is enrolled and kept in school it can delay her age at first marriage. Interventions to stop child marriage should therefore include a component that aims at improving retention of adolescent girls in school. With out of school adolescent girls, conscious efforts should be made to empower them through vocational skills building for them to be able to earn a living.

Participants in the interviews also noted that the police and other law enforcement institutions should step up efforts to curb child marriage. Hence, law enforcement agencies should put major focus on implementing and enforcing existing laws governing child marriage in Ghana. Some participants also indicated that chiefs should put in more efforts by speaking publicly against child marriage and make local decrees prohibiting child marriage, as well as penalise offenders.

### Limitation

A potential limitation of this study is that the qualitative study was conducted in selected districts in the Northern and Central regions; hence, the results cannot be generalized to the whole country. However, these findings give some indications of and reflect the issues surrounding child marriage in these areas. These findings might not be very different from what is experienced in other parts of the country.

## Conclusions

The findings reveal that various socio-economic and cultural factors such as education, teenage pregnancy, poverty and the exchange of girls for marriage influence child marriage. Hence, efforts to curb child marriage should be geared towards retention of girls in school, empowering girls economically through vocational training, enforcing laws on child marriage in Ghana, as well as designing tailored advocacy programs to educate key stakeholders and adolescent girls on the consequences of child marriage. Further, there is the need to address socio-cultural norms/practices to help end child marriage. Additionally, efforts should be directed towards curbing teenage pregnancy, which will lead to reducing child marriage. This could be done through working with reproductive health partners, both local and international, to improve adolescent girls’ access to and utilization of reproductive health services including family planning.

## Supplementary information

**Additional file 1: Appendix A.** Focus Group Discussion guides.

**Additional file 2: Appendix B.** Key Informant Interview guide.

## Data Availability

The survey and dataset used for this study were from the 2014 Ghana Demographic Health Survey. The survey is available in the report [27]. The quantitative dataset generated and/or analysed during the current study is also available on the Demographic and Health Surveys Program repository, https://dhsprogram.com/data/new-user-registration.cfm. The qualitative interview guides used in this study were developed for this study. Four FGD guides and one KII guide were utilized to elicit qualitative data as described in the Methods section (see page 10). The qualitative dataset generated and/or analysed during the current study are not publicly available due to funding agreements but are available from Population Council and UNFPA (info.ghana@popcouncil.org) upon written request and approval.

## Abbreviations

CEDAW: Convention on the Elimination of all Forms of Discrimination Against Women
DHS: Demographic and Health Survey
FGD: Focus Group Discussion
GHDS: Ghana Demographic Health Survey
KII: Key Informant Interviews
