# Supplementary material for: Child marriage in Ghana: evidence from a multi-method study
Source: BMC Womens Health. 2019 Nov 12;19:126. doi: 10.1186/s12905-019-0823-1 (PMC6833172; doi:10.1186/s12905-019-0823-1)
Supplement: Supplementary file 1 — Additional file 1: Appendix A. Focus Group Discussion guides. [file 12905_2019_823_MOESM1_ESM.docx]

## 1: Focus Group Discussion Guide for Married Adolescents and Young Women (12-24 years)

**FGD Details**

1. Facilitator’s Name: ____________________________
2. Note taker’s name: ____________________________
3. Number of FGD participants: _______________
4. Age group: ______________________
5. Location of FGD: ____________________________ Central [ ] Northern [ ]
6. Date of FGD (DD/MM/YYYY): _______/________/____________
7. Start time: ____________End time: ____________
8. Notes/Comments:_________________________________________________________________________________________________________________________________________________________________________________________________________

**Annex 9: Demographic Information of FGD Participants**

| **P** | **Age** | **Age at marriage** | **Edu.**  **level** | **Ethnic Group** | **Religion** | **Type of marriage** | **Living Arrangement** | **Number of children** | **Age of partner** | **Occupation of partner** |
| --- | --- | --- | --- | --- | --- | --- | --- | --- | --- | --- |
| 1 |  |  |  |  |  |  |  |  |  |  |
| 2 |  |  |  |  |  |  |  |  |  |  |
| 3 |  |  |  |  |  |  |  |  |  |  |
| 4 |  |  |  |  |  |  |  |  |  |  |
| 5 |  |  |  |  |  |  |  |  |  |  |
| 6 |  |  |  |  |  |  |  |  |  |  |
| 7 |  |  |  |  |  |  |  |  |  |  |
| 8 |  |  |  |  |  |  |  |  |  |  |
| 9 |  |  |  |  |  |  |  |  |  |  |
| 10 |  |  |  |  |  |  |  |  |  |  |

**Legend: *Education:*** 0= no education; 1= Primary; 2= JHS; 3= SHS; 4= Tertiary ***Type of marriage:*** 1= Monogamous; 2= Polygamous ***Living Arrangement***: 1= Marital; 2= Parental 3= Duolocal **Religion**: 0 = No religion; 1= Christianity; 2= Islam; 3= Other religion

***Annex 9: Focus Group Discussion Guide for Married Adolescents (12-14 & 15-17 year olds) and Young Women (18-24 year olds)***

**Thank you again for agreeing to participate in this research on child marriage and how you have experienced it, and how it is dealt with in your area. We will start by asking the first question:**

1. What are the reasons why girls in this area can get married before 18 years? ***Probe: Factors such as poverty, cultural practices, teenage pregnancy, school dropout, etc.***
2. Was it you who decided to get married or did someone else decide for you? **Probe: if someone else, probe who? Probe for differences, if any, between who initiates and who makes the final decision.**
   1. If girl herself: what were your reasons for wanting to get married early?
   2. If other: did anyone talk to you about the reasons to get married early? **Probe: parents, grandparents, community elders, teachers**? What were the reasons?
      1. What did you think/feel about the reasons they gave you?
      2. Did you feel you would be punished if you refused the reasons given to you?
      3. Would these punishments be justified?
3. Did any ceremony happen to show that you are now married? **Probe: traditional marriage ceremony with bride price; religious ceremony; verbal family exchange etc.**
4. What has changed in your life because you are now married?
   1. What has remained the same in your life because you are now married? **Probe: residence; schooling; societal recognition; empowerment; workload; childbearing; relationship with spouse, parents, community; freedom of movement; decision-making**
5. Among these things that you have mentioned (Q4), which of them have been good for you (benefits)?
   1. And which of them have been difficult to manage (challenges)?
6. Are you being given enough support as young married girls and women?
   1. Who provides this support?
   2. Do you find that it is sufficient enough to handle being young and married?
7. If your sister or your friend want to get married at the same age you got married, what advice will you give them? ***Probe: What have you learned from getting married early?***
8. Are some people saying child marriage should continue?
   1. What do you think will make it continue?
9. Are some people saying child marriage should stop?
   1. What do you think will make it stop? **Probe: higher bride price, legal action, educating the girl child**
   2. Who can make it stop? ***Probe: Girls themselves, family, community, governmental or non-governmental organisation(s)?***
   3. At what age do you think is appropriate for a girl to get married?
10. Do you know of any programs, policies or laws in Ghana about child/early marriage***? Probe: which ones and what they are about(if known)***
11. Is there any other issue related to child marriage that we have not brought up in this discussion yet? ***Probe: Encourage further discussions.***

**This is the end of the interview. Thank you for your time!**

## 2: Focus Group Discussion Guide for Unmarried Adolescents (12-17 year olds)

**FGD Details**

1. Facilitator’s Name: __________________
2. Note taker’s name: _____________________
3. Number of FGD participants: _______________
4. Location of FGD: _______________ Central [ ] Northern [ ]
5. Date of FGD (DD/MM/YYYY): _______/________/____________
6. Start time: ____________End time: ____________
7. Notes/Comments:_________________________________________________________________________________________________________________________________________________________________________________________________________

**Annex 10: Demographic Information of FGD Participants (Unmarried Adolescents, 12-17 year olds)**

| **Participant** | **Age** | **Educational level** | **Ethnic Group** | **Religion** | **Number of children** | **Occupation** |
| --- | --- | --- | --- | --- | --- | --- |
| 1 |  |  |  |  |  |  |
| 2 |  |  |  |  |  |  |
| 3 |  |  |  |  |  |  |
| 4 |  |  |  |  |  |  |
| 5 |  |  |  |  |  |  |
| 6 |  |  |  |  |  |  |
| 7 |  |  |  |  |  |  |
| 8 |  |  |  |  |  |  |
| 9 |  |  |  |  |  |  |
| 10 |  |  |  |  |  |  |

***Education:*** 0= no education; 1= Primary; 2= JHS; 3= SHS; 4= Tertiary;
**Religion**: 0 = No religion; 1= Christianity; 2= Islam; 3= Other religion

***Annex 10: Focus Group Discussion Guide for Unmarried Adolescents (12-17 year olds)***

**Preamble: Thank you again for agreeing to participate in this research on child marriage. As you may know, there are some girls in Ghana and around the world who get married early, either by their own choice or because they are forced to do so.**

- **Remember to emphasise NEUTRALITY !!**

1. What comes to your mind when we say child marriage?
   1. What are the reasons why girls in this area or in Ghana get married early? ***Probe: poverty, cultural practices, teenage pregnancy, school dropout, freedom, etc.***
2. Who normally decides that a girl will get married before 18 years?
3. Do you have any friends (or know of anyone) who got married before their 18^th^ birthday?
4. As young girls, do you feel forced to get married before 18 years? **Probe: community, family, government, church, mosque, and/or school**
5. What are the benefits of getting married early? ***Probe: girls themselves, their parents/guardians or significant others, etc.***
6. What are the problems with getting married early? ***Probe: Early child bearing, pregnancy complications, child or mother death, rape, school dropout, controlling husbands (movement, opinions, communication with opposite sex, decision making)***
7. Do you plan on waiting after your 18^th^ birthday to get married?
   1. If yes, what are your reasons for waiting until you pass the age of 18 years?
   2. Around what age do you think you will get married?
   3. If yes, what do you think are the benefits to delaying marriage?
   4. If yes, can there also be problems to delaying marriage?
   5. Who or what has influenced you to delay marriage until you are ready? **Probe: community, family, government, church, mosque, and/or school**
8. Are you being given enough support as young adolescents to delay marriage?
   1. Who provides this support?
   2. Do you find that it is sufficient enough to successfully delay marriage?
9. Are some people saying child marriage should continue?
   1. What do you think will make it continue?
10. Are some people saying child marriage should stop?
    1. What do you think will make it stop? **Probe: higher bride price, legal action, educating the girl child**
    2. Who can make it stop? ***Probe: Girls themselves, family, community, governmental or non-governmental organisation(s)?***
    3. At what age do you think is appropriate for a girl to get married?
11. Do you know of any programs, policies or laws in Ghana about child/early marriage? ***Probe: which ones and what they are about(if known)***
12. Is there any other issue related to child marriage that we have not brought up in this discussion yet? ***Probe: Encourage further discussions***

________________________________________________________________

- ***PLEASE EXCUSE 12-14 year olds here !!!!!!!!!!!!!!!!!!!!!!!!!!!!!!!!!!!!!!!!!!!!!!!!!!!!!***

1. [**ASK ONLY TO UNMARRIED 15-17 YEAR OLDS**] What kind of sexual and reproductive health services are available to adolescents and young women like you in this community? ***Probe: contraceptives, abortion, HIV and STI counselling and testing***
   1. How easy it to get these services?

**This is the end of the interview. Thank you for your time!**

## 3: Focus Group Discussion Guide for Unmarried Young Women (18-24 year olds)

**FGD Details**

1. Facilitator’s Name: __________________
2. Note taker’s name: _____________________
3. Number of FGD participants: _______________
4. Location of FGD: _______________ Central [ ] Northern [ ]
5. Date of FGD (DD/MM/YYYY): _______/________/____________
6. Start time: ____________End time: ____________
7. Notes/Comments:_________________________________________________________________________________________________________________________________________________________________________________________________________

**Annex 11: Demographic Information of FGD Participants (Unmarried Young Women, 18-24 year olds)**

| **Participant** | **Age** | **Educational level** | **Ethnic Group** | **Religion** | **Number of children** | **Occupation** |
| --- | --- | --- | --- | --- | --- | --- |
| 1 |  |  |  |  |  |  |
| 2 |  |  |  |  |  |  |
| 3 |  |  |  |  |  |  |
| 4 |  |  |  |  |  |  |
| 5 |  |  |  |  |  |  |
| 6 |  |  |  |  |  |  |
| 7 |  |  |  |  |  |  |
| 8 |  |  |  |  |  |  |
| 9 |  |  |  |  |  |  |
| 10 |  |  |  |  |  |  |

***Education:*** 0= no education; 1= Primary; 2= JHS; 3= SHS; 4= Tertiary;
**Religion**: 0 = No religion; 1= Christianity; 2= Islam; 3= Other religion

***Annex 11: Focus Group Discussion Guide for Unmarried Young Women (18-24 year olds)***

**Preamble: Thank you again for agreeing to participate in this research on child marriage. As you may know, there are some girls in Ghana and around the world who get married early, either by their own choice or because they are forced to do so.**

- **Remember to emphasise NEUTRALITY !!**

1. What comes to your mind when we say child marriage?
   1. What are the reasons why girls in this area or in Ghana get married before 18 years? ***Probe: poverty, cultural practices, teenage pregnancy, school dropout, freedom, etc.***
2. Who normally decides that a girl will get married before 18 years?
3. Do you have any friends (or know of anyone) who got married before their 18^th^ birthday?
4. As young girls, do you feel pressured to get married before 18 years? **Probe: community, family, government, church, mosque, and/or school**
5. Did you plan purposely not to get married before your 18^th^ birthday?
   1. If yes: what were your reasons/motivations/influences for delaying marriage?
   2. If yes: at what age do you plan on getting married? Why?
   3. If no: at what age would you have wanted to get married? Why?
6. As things are now, do you feel you have benefitted from waiting to get married?
   1. If so, how have you benefitted?
   2. What would your life look like if you had gotten married earlier?
7. Have you faced any problems because you have delayed marriage? ***Probe: Delayed child bearing, pregnancy complications, shrinking marriage market, etc.***
8. Are you being given enough support as young women to get married at the age you desire?
   1. Who provides this support?
   2. Do you find that it is sufficient enough to successfully delay marriage?
9. Are some people saying child marriage should continue?
   1. What do you think will make it continue?
10. Are some people saying child marriage should stop?
    1. What do you think will make it stop? **Probe: higher bride price, legal action, educating the girl child**
    2. Who can make it stop? ***Probe: Girls themselves, family, community, governmental or non-governmental organisation(s)?***
    3. At what age do you think is appropriate for a girl to get married?
11. Do you know of any programs, policies or laws in Ghana about child/early marriage***? Probe: which ones and what they are about(if known)***
12. Is there any other issue related to child marriage that we have not brought up in this discussion yet? ***Probe: Encourage further discussions***
13. What kind of sexual and reproductive health services are available to young women like you in this community? ***Probe: contraceptives, abortion, HIV and STI counselling and testing***
    1. How easy it to get these services?

**This is the end of the interview. Thank you for your time!**

## 4: Focus Group Discussion Guide for Parents & Other Adults in the Community

**FGD Details**

1. Facilitator’s Name: __________________
2. Note taker’s name: _____________________
3. Number of FGD participants: _______________
4. Location of FGD: _______________ Central [ ] Northern [ ]
5. Date of FGD (DD/MM/YYYY): _______/________/____________
6. Start time: ____________End time: ____________
7. Notes/Comments:_________________________________________________________________________________________________________________________________________________________________________________________________________

**Annex 12: Demographic Information of FGD Participants (Parents/Guardians/Gatekeepers)**

| **P** | **Sex** | **Age** | **Age at first marriage** | **Educational level** | **Marital status** | **Ethnic Group** | **Religion** | **Occupation** | **Number of children** |
| --- | --- | --- | --- | --- | --- | --- | --- | --- | --- |
| 1 |  |  |  |  |  |  |  |  |  |
| 2 |  |  |  |  |  |  |  |  |  |
| 3 |  |  |  |  |  |  |  |  |  |
| 4 |  |  |  |  |  |  |  |  |  |
| 5 |  |  |  |  |  |  |  |  |  |
| 6 |  |  |  |  |  |  |  |  |  |
| 7 |  |  |  |  |  |  |  |  |  |
| 8 |  |  |  |  |  |  |  |  |  |
| 9 |  |  |  |  |  |  |  |  |  |
| 10 |  |  |  |  |  |  |  |  |  |

**Legend: *Education:*** 0= no education; 1= Primary; 2= JHS; 3= SHS; 4= Tertiary ***Marital status:*** 1= Married; 2= Single; 3=divorced/separated; **Religion**: 0=no religion; 1=Christianity; 2=Islam; 3=Other religions

**Thank you again for agreeing to participate in this research on child marriage and how you have experienced it, and how it is dealt with in your area.**

- **Remember to emphasise NEUTRALITY !!**

1. In this community, who is considered a child?
2. What comes to your mind when we say child marriage?
   1. Is child marriage commonly practiced in this area?
3. Who usually decides or allows for girls to get married before 18 years?
4. What are the reasons why girls in this area can get married before 18 years? ***Probe: poverty, cultural practices, teenage pregnancy, school dropout, etc.***
5. Did anyone in this group get married before 18 years of age?
6. (*For those who affirm Q5*): Did you decide to get married at an early age or was it someone else’s decision? **Probe: if someone else, probe who? Probe for differences, if any, between who initiates and who makes the final decision.**
7. What circumstances led to the decision for you to get married?
8. What are the benefits that come from early/child marriage for a girl and her family? ***Benefits for girls themselves, their parents/guardians or significant others, etc.***
   1. What have been the benefits of **you** getting married at an early age?
9. What are the problems that come from early/child marriage for a girl and her family? ***Early child bearing, pregnancy complications, child or mother death, rape, school dropout, controlling husbands (movement, opinions, communication with opposite sex, decision making)***
   1. What have been the problems with **you** getting married at an early age?
10. What happens if a girl and/or her family refuse early marriage? Are these consequences justified?
11. How do parents/guardians/community members usually encourage or promote child marriage? ***Probe: Are there any incentives?***
12. How do parents/guardians/community members usually discourage or prevent child marriage?

**Perspectives on Laws, Policies and Programmes to address Child Marriage**

1. Are some people saying child marriage should continue?
   1. What do you think will make it continue?
2. Are some people saying child marriage should stop?
3. What do you think will make it stop? **Probe: higher bride price, legal action, educating the girl child**
4. Who can make it stop? ***Probe: Girls themselves, family, community, governmental or non-governmental organisation(s)?***
5. At what age do you think is appropriate for a girl to get married?
6. Do you know of any programs, policies or laws in Ghana about child/early marriage***? Probe: which ones and what they are about (if known)***
7. Is there any other issue related to child marriage that we have not brought up in this discussion yet? ***Probe: Encourage further discussions.***

**This is the end of the interview. Thank you for your time!**
