# Supplementary material for: Child marriage in Ghana: evidence from a multi-method study
Source: BMC Womens Health. 2019 Nov 12;19:126. doi: 10.1186/s12905-019-0823-1 (PMC6833172; doi:10.1186/s12905-019-0823-1)
Supplement: Supplementary file 2 — Additional file 2: Appendix B. Key Informant Interview guide. [file 12905_2019_823_MOESM2_ESM.docx]

## 1: Key Informant Interview Guide for Focal Persons & Key Stakeholders

Informant’s Name: ________________________________________________________

Informant’s Position: _______________________________________________________

Informant’s Organization/Institution: __________________________________________

Organization/Institution primary area of focus: ___________________________________

Length of time focal person/stakeholder has worked with institution (in years):___________

Interview Date: ____________________________

Interviewer’s Name: ___________________________

Start time: ____________End time: ____________

**Thank you again for agreeing to participate in this research on child marriage and how you have experienced it, and how it is dealt with in your area. We will start by asking the first question:**

1. What activities or programs is your ministry/department/organization engaged in to address the health of adolescent girls and young women in Ghana?
2. In your opinion, how widespread is the practice of child marriage in Ghana?
   1. In this region?
   2. In this area? [**Probe: in this region, higher or lower than national average (of 21% in 2014 and 25% in 2008?)**]
3. What has your ministry/department/organization found to be the main drivers of or reasons for child marriage in Ghana?
   1. In this region?
   2. In this area? ***Probe: Factors such as poverty, cultural practices, teenage pregnancy, school dropout, etc.***
4. What specific actions has your ministry/department/organization taken to address the problem of child marriage in Ghana?
   1. In this region?
   2. In this area? ***Probe: Please describe these actions in detail.***
5. Has your ministry/department/organization partnered with another to address the problem of child marriage in Ghana?
   1. In this region?
   2. In this area? ***Probe: If yes, describe collaboration; if not, describe reasons.***
6. How is your ministry/department/organization addressing child marriage with stakeholders and gatekeepers are keen on promoting child marriage?
7. Who have your your ministry/department/organization found to be community champions for ending child marriage?
   1. How are they being equipped to support the cause?

**Perspectives on Laws, Policies and Programmes to address Child Marriage**

1. What is your opinion on the current programs, policies, and/or laws in Ghana that are aimed to end child marriage?
   1. Which ones are effective and which need strengthening/revision? **Probe: please discuss your reasons**.
2. Is there any other issue related to child marriage that we have not brought up in this interview yet?

**This is the end of the interview. Thank you for your time!**
